# Sex and apolipoprotein E (APOE) genotype Interact to impact relationships among sleep, Alzheimer's disease biomarkers, and memory in older adults

**DOI:** 10.1002/alz70855_105374

**Published:** 2025-12-24

**Authors:** Negin Sattari, Abhishek Dave, Kitty K. Lui, Kate E Sprecher, Miranda G. Chappel‐Farley, Ivy Y. Chen, Brady A Riedner, Barbara B. Bendlin, Gwendlyn Kollmorgen, Clara Quijano‐Rubio, Henrik Zetterberg, Kaj Blennow, Bryce A Mander, Ruth M Benca

**Affiliations:** ^1^ Department of Psychiatry and Human Behavior, University of California, Irvine, Irvine, CA, USA; ^2^ Department of Cognitive Sciences, University of California, Irvine, Irvine, CA, USA; ^3^ SDSU / UC San Diego Joint Doctoral Program in Clinical Psychology, San Diego, CA, USA; ^4^ University of Wisconsin, Madison, WI, USA; ^5^ University of California, Irvine, Irvine, CA, USA; ^6^ University of Wisconsin Madison Psychiatry, Madison, WI, USA; ^7^ Department of Psychiatry, University of Wisconsin School of Medicine and Public Health, Madison, WI, USA; ^8^ University of Wisconsin ‐ Madison, Madison, WI, USA; ^9^ Wisconsin Alzheimer's Institute, University of Wisconsin‐Madison, Madison, WI, USA; ^10^ Wisconsin Alzheimer's Disease Research Center, University of Wisconsin‐Madison, School of Medicine and Public Health, Madison, WI, USA; ^11^ Roche Diagnostics GmbH, Penzberg, Germany; ^12^ Roche Diagnostics International Ltd., Rotkreuz, Switzerland; ^13^ University of Gothenburg, Mölndal, Gothenburg, Sweden; ^14^ University of Gothenburg, Mölndal, Sweden; ^15^ Center for the Neurobiology of Learning and Memory, University of California, Irvine, Irvine, CA, USA; ^16^ Wake Forest School of Medicine, Wake Forest University, Winston‐Salem, NC, USA

## Abstract

**Background:**

Alzheimer's disease (AD) is characterized by tau and amyloid‐β (Aβ) pathologies, influenced by factors such as sex, aging, APOE genotype, and sleep. Sleep is essential for memory consolidation, but its interaction with these factors and AD biomarkers remains unclear. This study examined how sex, APOEε4 status, and CSF AD biomarkers (Aβ42/40, *p*‐tau) affect the relationship between sleep and overnight memory retention in older adults.

**Method:**

Sixty‐six Wisconsin Registry for Alzheimer's Prevention participants (41 women, 25 ε4‐carriers, mean age 61.8±6.1 years) encoded word pairs in the evening, underwent overnight polysomnography, and completed a morning memory test. Memory retention was calculated as the morning‐evening difference, with sleep stages quantified. CSF biomarkers (*p*‐tau, Aβ42/40) were assessed using the exploratory NeuroToolKit panel on Cobas® analyzers (Roche Diagnostics International Ltd, Rotkreuz, Switzerland) under strict quality control. Moderated mediation analysis (PROCESS model 11) examined relationships between sleep stages (predictor), memory retention (outcome), biomarkers (mediators), and sex/APOEε4 status (moderators).

**Result:**

A sex×APOEε4 interaction predicted memory (*p* = 0.02) where female ε4‐non‐carriers exhibited more forgetting than ε4‐carriers (*p* = 0.02). Male ε4‐carriers had worse memory retention than female ε4‐carriers (*p* = 0.01). Female ε4‐carriers had more N3‐sleep stage (*p* = 0.01), correlating with better memory retention (r=0.72, *p* = 0.02), while male ε4‐carriers had more N2‐sleep (*p* = 0.04), also linked to better memory retention (r=0.68, *p* = 0.03). Moderated mediation analysis demonstrated that N2‐sleep negatively predicted *p*‐tau levels across participants (*p* = 0.03), suggesting a potential protective effect, while higher *p*‐tau was linked to lower overnight memory retention (*p* = 0.04). A significant interaction between sex and APOEε4 (*p* = 0.05), N2 and APOEε4 (*p* = 0.07‐trend), and a main effect of APOEε4 (*p* = 0.04) on Aβ42/40 levels suggest that APOEε4 status is a determinant of Aβ42/40. The findings indicate that the relationship between N2‐sleep and Aβ42/40 is not uniform but are moderated by whether an individual carries the APOEε4 allele. No significant associations were found with N3‐sleep (*p* > 0.05).

**Conclusion:**

Our findings show that NREM sleep stages influence overnight memory retention in older men and women, depending on APOE genotype and CSF AD biomarkers, suggesting that sex differences in long‐term memory retention are shaped by interactions between sleep and AD pathology.

